# Conformity cannot be identified based on population-level signatures

**DOI:** 10.1038/srep36068

**Published:** 2016-10-31

**Authors:** Alberto Acerbi, Edwin J. C. van Leeuwen, Daniel B. M. Haun, Claudio Tennie

**Affiliations:** 1 Eindhoven University of Technology, School of Innovation Sciences, Eindhoven, 5600 MB, The Netherlands; 2 University of St Andrews, School of Psychology & Neuroscience, Westburn Lane, St Andrews, Fife, KY16 9JP, United Kingdom; 3 Max Planck Institute for Psycholinguistics, Wundtlaan 1, 6525 XD Nijmegen, The Netherlands; 4 University of Leipzig, Department of Early Child Development and Culture and Leipzig Research Center for Early Child Development, Jahnallee 59, Leipzig, 04109, Germany; 5 University of Birmingham, School of Psychology, Edgbaston, Birmingham, B15 2TT, United Kingdom

## Abstract

Conformist transmission, defined as a disproportionate likelihood to copy the majority, is considered a potent mechanism underlying the emergence and stabilization of cultural diversity. However, ambiguity within and across disciplines remains as to how to identify conformist transmission empirically. In most studies, a population level outcome has been taken as the benchmark to evidence conformist transmission: a sigmoidal relation between individuals’ probability to copy the majority and the proportional majority size. Using an individual-based model, we show that, under ecologically plausible conditions, this sigmoidal relation can also be detected without equipping individuals with a conformist bias. Situations in which individuals copy randomly from a fixed subset of demonstrators in the population, or in which they have a preference for one of the possible variants, yield similar sigmoidal patterns as a conformist bias would. Our findings warrant a revisiting of studies that base their conformist transmission conclusions solely on the sigmoidal curve. More generally, our results indicate that population level outcomes interpreted as conformist transmission could potentially be explained by other individual-level strategies, and that more empirical support is needed to prove the existence of an individual-level conformist bias in human and other animals.

Conformist transmission is considered a potent mechanism underlying the emergence and stabilization of human cultural diversity. It has been shown, by means of formal modelling, that conformist transmission can facilitate and safeguard cultural variation from erosion toward similarity, although it may not be the only mechanism that can do so^1^,^2^. Such stable cultural variation, in turn, has been proposed as a prerequisite for cultural selection between groups, claimed to be the necessary factor to explain the extraordinary range of cooperation and prosociality in the human species^3^,^4^,^5^. At the same time, claims of “conformity” have recently been reported in a diversity of non-human animal species, such as “conformity” in rats^6^; chimpanzees^7^,^8^; vervet monkeys^9^; “conformist transmission” in sticklebacks^10^; and great tits^11^ (see refs 12 and 13 for review).

Despite its importance, conformist transmission has been defined in numerous, often incompatible, ways. For instance, “conformity” has been equated with social influence trumping personal knowledge (see ref. 14), irrespective of majority considerations (see refs 15 and 16). Notably, the presence of “conformity” has been claimed in scenarios where individuals actually adopt the behaviour of the majority^9^, but this outcome is expected ‘almost any time there is cultural transmission’^1^, and can simply be instantiated by individuals copying randomly (ref. 1, also see ref. 17). Overall, an extensive source of confusion regarding conformity definitions is that some of them refer to population-level outcomes (henceforth “PLOs”; e.g., “behavioural homogeneity”), while others refer to individual-level strategies (henceforth “ILSs”; e.g., “copy the majority”).

Cultural evolution models adopt a precise definition of conformist transmission, which entails individuals having a *disproportionate* tendency to copy the majority (henceforth “conformist bias”). This means that, to show a conformist bias, an individual should have a probability to copy the majority that is *higher* than the proportion of the majority itself. In other words, if 60% of individuals in a group show a behaviour A, a conformist individual should have a probability to copy A higher than 60%. Importantly, only this stricter version of conformist transmission has been shown, with formal models, to be sufficiently potent to maintain cultural diversity (see refs 1 and 2), and consequently enable cultural group selection to shape the extended forms of cooperation and pro-sociality characteristic of the human species^3^,^4^,^5^. This disproportionate tendency to copy the majority generates, at population level, a sigmoidal (or “S-shaped”) relation between the probability of copying the majority and the proportional majority size–henceforth “the sigmoid” (refs 1,2,17, 18, 19, 20, 21, see also examples in Fig. 1). However, while it follows that a conformist bias (ILS) will result in the sigmoid (PLO), it is in an open issue under which assumptions it is correct to infer the existence of a conformist bias (ILS) from the sigmoid (PLO). This question is particularly relevant as the empirical support for the existence of an individual-level conformist bias is not uncontroversial for humans (ref. 22, see ref. 23 for a recent review; also see Discussion below) and even more so for the recent claims of conformity in non-human animals (refs 24, 25, 26, 27 and Discussion below).

Here we investigated, with an individual-based model, if a sigmoidal relation between the probability of adopting a cultural variant and its proportional frequency in the population (i.e. the sigmoid PLO) could be detected in the absence of conformist bias. More specifically, mimicking socio-ecological conditions revolving around plausible learning trade-offs and mechanisms (e.g., kin- or group-biased copying, increasing conservatism with respect to adopting new variants, individual biases for a particular variant, etc.), we explored whether the sigmoid could be observed with individuals with learning tendencies different from a conformist bias.

We analysed 10 different conditions (see Table 1). Two of them (*Implicit knowledge* and *Rule of 3s*) are based on previous models of conformist transmission and explicitly implement a conformist bias, while another one (*Random copying*) by definition produces a linear relation between frequency of a variant and probability to copy. These conditions were included to validate our methodology. The other conditions represent various learning strategies^18^ that have been previously modelled in other contexts, or situations that are likely to be present in the experimental settings used to test the presence of “conformity”. The cumulative outcomes of copying events were collected for each condition, and they were used to generate the relation between frequency of variant and probability to copy. This function was then fitted with both a sigmoid model and a linear model, and the AICs of the two were calculated^28^. The difference between the two AICs gives an indication of how well the data are fitted by one or by the other model, with positive delta AICs indicating a better sigmoidal fit, and negative delta AICs indicating a better linear fit, i.e. indistinguishable from outcomes produced by unbiased copying^1^,^20^.

## Results

**Figure 1:**
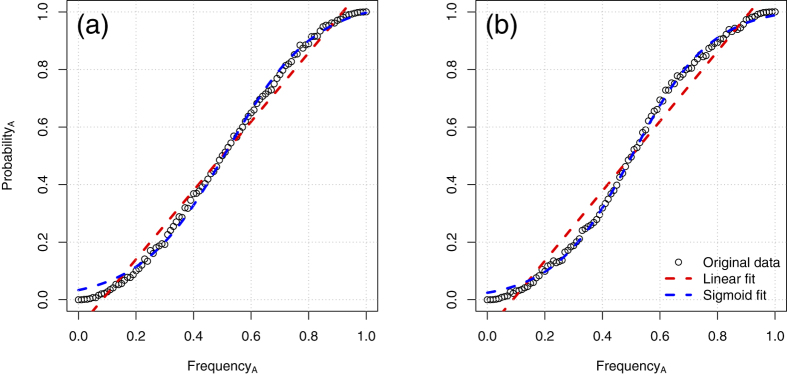
Conformist transmission rules producing sigmoid curves. (**a**) Individuals have a disproportionate tendency to copy the majority, and they know the variant distribution across the entire population (condition *Implicit knowledge*) or (**b**) individuals sample a subset of individuals of the population and copy the majority (condition *Rule of 3s; s = 3*). Copying probability is plotted against frequency of the variant in the population.

We first validated our model in the conditions *Implicit knowledge* and *Rule of 3s*. In the former condition, individuals have an implicit knowledge of the variants distribution in the population, and they adopt the majority variant with a probability greater than the proportional majority size^1^. In the latter condition, individuals sample a random subset from the population, and choose the most common variant among this subset^1^,^2^. As expected, both conditions revealed a better fit of the sigmoid model with respect to the linear model at population level (see Fig. 1, Δ AIC = 154.12 and Δ AIC = 258.13 respectively, with sample size *s* = 3 for the *Rule of 3s* condition). Increasing the size of the sampled population in the *Rule of 3s* condition generated more pronounced sigmoids (with *s* = 33, for example, Δ AIC = 620.328).

**Figure 2:**
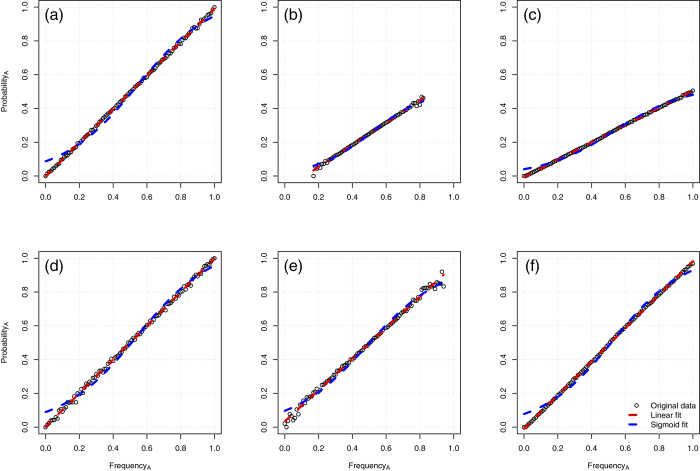
Conditions with better linear than sigmoid fit. (**a**) *Random copying* (Δ AIC = −393.51), (**b**) *Copiers Subgroup* (Δ AIC = −67.08, *Cp* = 50), (**c**) *Copy or not* (Δ AIC = −372.55, *pC* = 0.5), (**d**) *Family bias* (Δ AIC = −243.36, *S = *5), (**e**) *Information retention* (Δ AIC = −34.12, *m* = 3), (**f**) *Increasing conservatism* (Δ AIC = −360.3512, *d = *0.1). Copying probability is plotted against frequency of the variant in the population.

**Figure 3:**
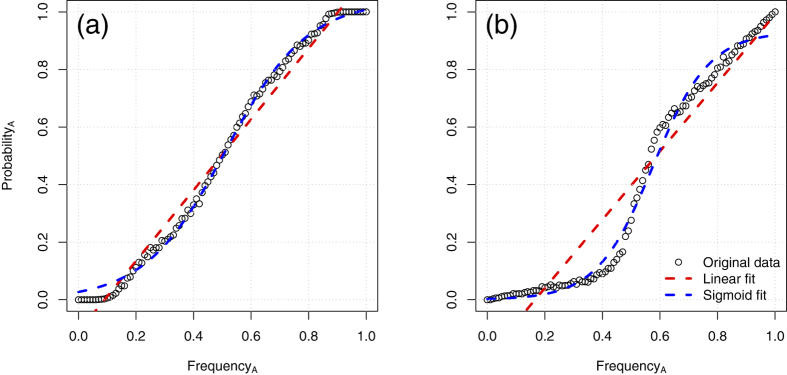
Conditions with better sigmoid than linear fit. Individuals (**a**) copy only a fixed subset of the population (condition *Demonstrators subgroup; Dm = *5) or (**b**) individuals have a lower preference for one of the two variants (condition *Variant Preference; pLess = *0.2). Copying probability is plotted against frequency of the variant in the population.

Of the remaining eight conditions, six were not better described by a sigmoidal relationship–instead, a linear model fitted the simulated data better for all the range of parameters tested (see Fig. 2 for representative examples and Δ AIC values). Two of the conditions, however, resulted in a better fit of the sigmoid, compared to a linear relationship: *Demonstrators subgroup* and *Variant Preference* (see Fig. 3, Δ AIC = 169.58 and Δ AIC = 199.65 respectively, with *dm* = 5 (number of demonstrators) for the *Demonstrators subgroup* condition, and *pLess* = 0.2 (strength of the preference for the less preferred variant) in the condition *Variant Preference*).

**Figure 4:**
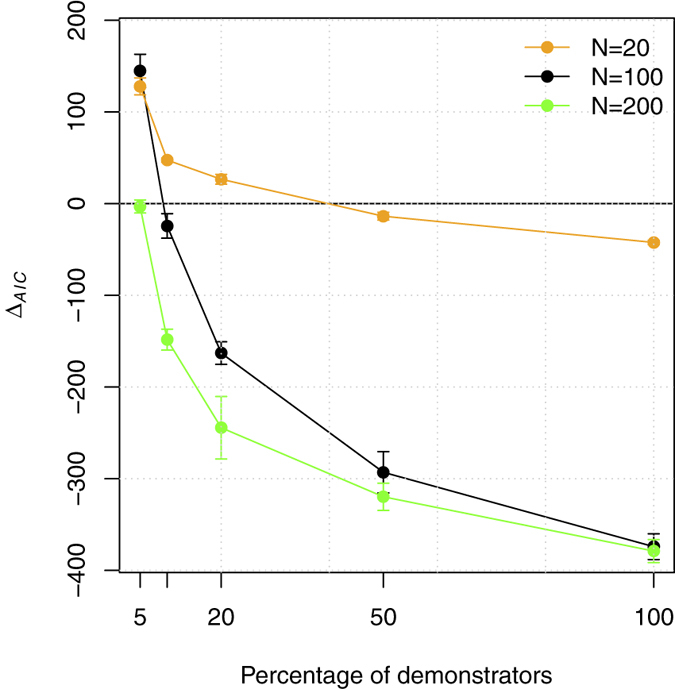
Sigmoid fit is more evident in small populations in the condition *Demonstrators subgroup*. The plot shows the relation between the proportional size of the subset of the population being copied (*Dm*) and the difference between AICs for sigmoid and linear fittings (positive values indicate that the sigmoid fitting is better). Simulations are run for *N* = 100 (the original set-up), and for smaller (*N* = 20) and bigger (*N* = 200) populations. Each data point represents the average of 10 experiments (each of them composed by 1,000 repetitions of the simulation). Errors bars show standard deviation.

In the case of the condition *Demonstrators subgroup,* only a fixed subset of individuals in the population provided the pool of demonstrators, and individuals copied randomly among them. The results show that, when the subgroup of demonstrators was represented by a small fraction of the population (5% when *N* = 100), the sigmoid PLOs provided a better fit than a linear model. Given this result, we ran additional simulations to investigate the outcomes of the condition *Demonstrators subgroup* with smaller (*N* = 20) and larger (*N* = 200) population sizes (original population size: *N* = 100). With small populations, conditions with relatively larger subgroups of demonstrators could still result in support for a sigmoid over a linear PLO (in the case of *N* = 20, 20% of individuals, see Fig. 4).

**Figure 5:**
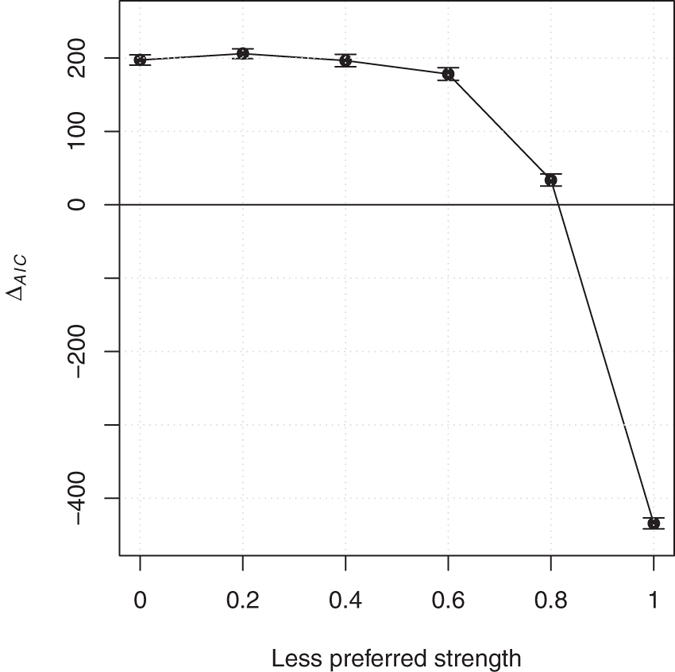
Sigmoid fit for various strengths of preferences in the condition *Variant preference*. Relation between the strength of the preference for the less preferred variant (*pLess*) –the preferred variant is always copied–and the difference between AICs for sigmoid and linear fittings (positive values indicate that the sigmoid fit is better). Each data point represents the average of 10 experiments (each of them composed by 1,000 repetitions of the simulation). Errors bars show standard deviation.

Finally, in the case of *Variant Preference*, individuals had a preference for one of the two variants, which was copied each time an individual was paired with a demonstrator showing it. The other variant was instead copied with a probability equal to the parameter *pLess*. Models results show that, aside from the case where *pLess* = 1, which is in fact equivalent to random copying, all values of *pLess* produced positive Δ AIC, i.e. a sigmoid function was fitting the data better than a linear one (see Fig. 5).

## Discussion

Our results show that, in realistic cases, even without endowing individuals with a conformist bias, a sigmoid relation between the frequency of a variant and the probability to copy the variant can appear more plausible than a linear one. These findings reveal a possible–and currently unaddressed–confound in conformity research: the fact that a conformist bias results in a sigmoidal pattern at the population level does not mean that, conversely, a sigmoidal pattern at the population level is always produced by a conformist bias at individual-level.

The conditions that supported the sigmoid fit in absence of conformist bias are ecologically relevant. For instance, model biases (e.g., copy experts or copy dominants) produce situations analogous to our *Demonstrator subgroup* condition (notice that our condition uses a random subset of a population, but this does not qualitatively change the results). Model biases have been documented across many animal species^7^,^29^,^30^ and might arguably require less cognitive effort to implement than a bias that is based on a mental representation of the distribution of traits across the population. Even less demanding, our *Demonstrator subgroup* condition could come about cognitively “for free” if–as highly plausible–some individuals are consistently more active, and hence more conspicuous as demonstrators, than others (e.g. ref. 31). The sigmoid is produced in this condition because any subset of the population–in our specific case, the group of demonstrators–will tend to be more homogeneous, on average, than the whole population (for the same reason as when one flips two coins one is more likely to produce the same result as when one flips more, e.g. ten, coins). Majority behaviours in this subset will thus be copied with a probability that is higher than their frequency in the whole population.

Similarly, content biases that underline our *Variant preference* condition seem almost unavoidable in real-life settings, and they pervade cultural transmission studies, even when there is an explicit attempt to avoid them (e.g., see “poke” vs. “lift” for chimpanzees in ref. 32; or “text” vs. “drawings” for humans in refs 33 and 34). Moreover, content biases may coincidentally arise as an effect of local/stimulus enhancement, for example when one of the variants becomes more conspicuous due to others’ actions leaving traces (e.g., if certain tools are left behind at a relevant location^35^,^36^). In this condition, the sigmoid fit is better than the linear because copying probability is a function *both* of the frequency of the variant *and* of how much the variant is preferred, and the two factors interact. In our simulations, at lower frequencies we find less preferred variants, which will tend to be copied even relatively less than their frequency (exactly because they are not preferred) producing a sigmoid relationship, while preferred variants will be found at higher frequencies, and they will always be copied, producing a linear relationship (see Fig. 3b). Overall, the combination of these two effects generates a nonlinear relation between frequency and probability to copy a variant that can be mistakenly taken as a sigmoid.

Our results highlight the fact that caution is warranted when the PLO sigmoid is presented as evidence of a conformity bias. Just as s-shaped curves in diffusion-curve analysis can no longer be taken as reliable indicators for social learning (e.g. ref. 37), we have shown that the classic conformist transmission sigmoid (where copy probability is plotted against frequency) is an unreliable indicator of conformity at the individual level. Our study identified two specific scenarios that conformity researchers (relying on the sigmoid) should be vigilant of: one in which individuals have a preference for one of the two cultural variants, and one in which not the entire population is sampled with equal probability, but instead a group of individuals within the population act as demonstrators for all others. We see these two scenarios as highly plausible, even in published accounts in which the better fit of the sigmoid compared to a linear relationship between frequency and copying probability is key to the conformity claim (e.g. ref. 11). In order to be able to identify conformity, based on our results, we would like to make two suggestions: (1) variant preferences should be investigated and controlled for, and (2) selective copying of individuals should be ruled out as alternative explanation.

The first suggestion can typically be taken into account by testing a control group of animals in which no demonstrators have been active (e.g. refs 8,11,24 and 32). If the control individuals do not show a preference for one or the other variant, then variant preferences are considered non-existent. Variant preferences, however, can also emerge in the context of the experiment. For instance, if a highly active individual engages in the experimental task by repetitively using one particular technique (e.g. sliding a door to the left), this technique can become easier or more salient to employ than the alternative one (e.g. by smoothening the sliding mechanism on the left side, denting the door at the location from which pushing to the left is easiest in terms of exerting force, leaving residues, or by systematically varying the starting position of the door, etc.). Consequently, subsequent individuals may be biased toward one of the two variants (in this case, the left sliding technique).

The second suggestion boils down to a scrutiny on the level of learning biases. We suggest that it is important to check for possible evidences that a subgroup of individuals (e.g. elders, or experts) is copied more than others, or that some individuals are consistently more active, and thus they will tend to be copied more. In these cases, it becomes imperative to scrutinize individual behavior, or, more specifically, code and analyze the observation records of all respective individuals^38^.

Given that most prior research is based on the assumption that conformist transmission can be evidenced by identifying the sigmoid (e.g. refs 10,11,34,39 and 40), we wonder whether we have, at the moment, strong support for the existence of conformist transmission at all. The conjecture that conformist bias as individual-level mechanism might not play an important role in the real lives of individuals is supported by recent critiques on the most promising accounts of conformity in non-human animals. For instance, in line with the results from our model, Heyes and Pearce^25^ suggest that conformist transmission in sticklebacks^10^ might be better explained by the simpler mechanism of paying selective attention to heightened foraging activity. Similarly, conformist transmission in great tits^11^ was alternatively explained in terms of simpler mechanisms like ‘copy when uncertain’ and/or ‘prefer social over individual information’^26^,^38^.

Even in humans, solid evidence for conformist transmission remains difficult to find^12^,^23^, or it is not as strong as the evidence for other commonly studied learning strategies^22^. Nevertheless, there are some convincing experimental accounts of human subjects responding in a conformist transmission fashion^20^,^41^,^42^,^43^. Interestingly, however, all these studies have in common the same mode of information acquisition for the subjects, which is instantaneous and cost-free. In other words, subjects are able to obtain instant knowledge of the frequency distribution of strategies across the population by merely looking at a computer screen, thereby bypassing the need for continuous effort (e.g., time, energy) and cognitive functioning (e.g., memory, information integration). Moreover, while these experiments with human subjects showed that the sigmoid curve was produced by a disproportionate individual-level tendency to copy the majority, the set-up of the experiments was often already “suggesting” this behaviour to the subjects (showing explicitly, for example, a small group of individuals in which some of them were proposing one option and others not, and then asking the subject which option to choose, see ref. 42). It is at least reasonable, given the possibility of obtaining similar outcomes with alternative strategies, to wonder how likely it is to expect conformist bias in more realistic scenarios. In fact, conformist transmission in human subjects remained elusive in real-life scenarios, despite focused attempts (see refs 39 and 44). More specifically, when frequency-related information is not the *only* source of information for individuals to base their decisions on–arguably more typical of real-life scenarios than the opposite–conformist bias seems to dissolve. For instance, when the suggestion is created that a small subgroup of the population is more knowledgeable than the majority, or when the minority option possesses more intrinsic value than the option chosen by the majority, human children do not copy the majority (see refs 45, 46, 47, 48). Note that both copying from a subset of the population, for example because the concerning individuals possess more expertise (i.e. ‘copy experts’), and intrinsically preferring one option over the other are exactly the individual-level strategies we proved in this study to result in the same population-level signature as the conformist bias.

While other studies have highlighted how simple individual-level mechanisms are able to create and maintain stable cultural diversity^49^,^50^,^51^,^52^, conformist bias in particular has been identified in formal models as a mechanism able to explain both cultural within-group stability and between-group diversity under a wide range of conditions, including evolutionary forces such as adaptive trade-offs, selection, and migration (e.g. see refs 1 and 2). Such an outcome has been a key feature of accounts of cultural group selection aimed at explaining the evolution and current existence of the extraordinary forms of cooperation and pro-sociality observed in the human species^53^. Given the findings of the current study, an exciting question for future research is whether such stable cultural diversity can be maintained–in the face of evolutionary pressures–*without* an individual-level conformist bias, possibly by the sigmoid-producing ILSs identified by our study–or by some of their combinations.

## Methods

### Model outline

We studied populations of *N* = 100 individuals which were randomly initialized with one of two possible variants, A and B. At each time step, each individual had the opportunity to copy another individual from the population, the demonstrator. In detail, individuals were extracted in sequential order from the population, and paired with a demonstrator (also extracted from the population), and they could copy or not. Their status was updated immediately, so that they could become demonstrators for other individuals in the same time step, reflecting the copying event. The rules according to which the selection of the demonstrator, and the choice whether to copy or not happened, depended on the specific condition (see below).

Simulations ended when one of the two variants reached fixation, or they were stopped after 100 time steps (corresponding to 10,000 possible individual interactions). The model outputs were based on 1,000 replications of each condition. In each replication we recorded, for each interaction, whether individuals copied or not, and the frequency of the trait in the population at that moment. Copying was considered successful when an individual displayed, after the interaction, the same variant of the demonstrator, independently of his previous status, and provided that copying happened as indicated in the condition. Thus, when there was a change of variant, copying was always considered successful, while when then variant remained the same, it depended on the condition. In the latter case, for example, in the *Random copying* condition (see below) all interactions were considered successful copying events, while in the *Copiers subgroup* condition (see below), interactions were considered successful copying events only when the individual was a member of the subset of the population allowed to copy (see below for descriptions of each condition).

The data collected were used to create the function linking the frequency of variant versus the probability to copy it for each condition. We then fitted this function with a sigmoid and a linear model, and we calculated the AICs of the two models. The difference between the two AICs provides a way to evaluate how well the data are fitted by one or by the other model, with positive delta AICs indicating that the sigmoidal fit is better than the linear fit, and vice versa. Codes of the simulations are available online^54^.

### Conditions

#### Implicit knowledge

In the original analytical formulation of conformist transmission, Boyd and Richerson derive an equation that tracks the frequency of a trait assuming frequency-dependent cultural transmission. We validated our model implementing an individual-based version of this rule, where individuals have implicit knowledge of the variants distribution in the population, and adopt the majority variant with a probability greater than the proportional majority size. At each time step, each individual assumed a new trait as a function of the state of the population, with probability given by Boyd and Richerson’s Equation 7.1 (1) with *D* = 1 (see Table 1 for a summary of this and all other conditions).

#### Rule of 3s

The equation above is derived assuming that individuals sample three demonstrators in the population. We mirrored this implementation by endowing individuals (instead of having explicit knowledge of the variants distribution in the entire population) with the capacity to sample a subset of random individuals from the population and always copying the most common variant among them. Again, we validated our model using this rule, testing different samplings of the population (*s* = 3,5,9,17,33).

#### Random copying

At each time step, each individual was paired with a random demonstrator and it always copied it. This ILS implements the learning rule conventionally referred to as “unbiased transmission”^1^,^2^ or “random copying”^55^.

#### Copiers subgroup

The proportion of individuals in the population who adopted social information was varied. At the beginning of each repetition, a subset *S* of individuals in the population, of size *Cp* (*Cp* = 5,10,20,50), was randomly chosen. At each time step, each individual was paired with a random demonstrator, but only individuals belonging to the subset *S* copied the variant of the demonstrator. All other individuals retained the variant they already possessed. This condition corresponds to individual differences in learning tendencies^56^,^57^.

#### Demonstrators subgroup

The proportion of individuals in the population who served as demonstrators was varied. At the beginning of each repetition, a subset *S* of individuals in the population, of size *Dm* (*Dm* = 5,10,20,50), was randomly chosen. At each time step, each individual was paired with a demonstrator chosen from the subset *S* and always copied it. Demonstrators themselves changed their state according to the same rule. This condition corresponds to demonstrator biases like copy dominants or experts^7^,^58^.

#### Copy or not

The probability of copying varied according to a parameter *pC* (*pC* was varied continuously between 0 and 1). At each time step, each individual was paired with a randomly chosen demonstrator and it copied according to *pC.* Individuals that did not copy retained the variant they already possessed.

#### Family bias

Individuals copied only within subgroups/families, thus each individual copied within a different subgroup of population members. At the beginning of each repetition, the population was randomly divided in subgroups of size *S* (*S* = 5,10,20,50). At each time step, each individual was paired with a demonstrator selected from its subgroup and always copied it. This condition corresponds to demonstrator biases, specifically copy kin^59^,^60^,^61^.

#### Information retention

Individuals retained sampling information from previous rounds. At each time step, each individual was paired with a random demonstrator, and it added to its “memory slot” of size *m* (*m* = 3,5,10) the information on the variant possessed by the demonstrator. Individuals then chose randomly a variant within their memory. This condition corresponds to a random, or unbiased, copying model with memory^62^.

#### Variant preference

Individuals had a preference for one of the two variants, which was operationalized by endowing individuals with different copying probabilities for the two variants. When presented with a demonstrator showing it, one of the variant was always copied, while the other variant (the less preferred) was copied according to the parameter *pLess* (*pLess* was varied between 0 and 1). All individuals preferred the same variant, which was randomly selected at the beginning of each repetition. This condition corresponds to a content bias, and could also be resulting from local/stimulus enhancement (ref. 63, see Discussion).

#### Increasing conservatism

All individuals were set to learn socially, but with each time step a smaller proportion of the population copied, compared to retaining the previous variant. The pace of the decrease in probability to copy was set linear with time (i.e. at time step = 0 all individuals were social learners, and at time step = 100 all were individual learners), and the decrease affected a proportion *d* (*d* = 0.1,0.2,0.3) of the population. This model condition corresponds to a habit formation/conservatism bias^24^,^64^,^65^.

## Additional Information

**How to cite this article**: Acerbi, A. *et al*. Conformity cannot be identified based on population-level signatures. *Sci. Rep.* **6**, 36068; doi: 10.1038/srep36068 (2016).

**Publisher’s note:** Springer Nature remains neutral with regard to jurisdictional claims in published maps and institutional affiliations.

## Figures and Tables

**Table 1:** Summary of model conditions.

Condition name	Parameter	Sigmoid?	Additional tests
**Implicit knowledge**	***D*=1**	**Yes**	
**Rule of 3s**	***s*=3,5,9,17,33**	**Yes**	
Random copying		No	
Copiers subgroup	*Cp*=5,10,20,50	No	
**Demonstrators subgroup**	***Dm*=5,10,20,50**	**Yes**	**N=20,200**
Copy or not	*pC*=[0,1]	No	
Family bias	*S*=5,10,20,50	No	
Information retention	*m*=3,5,10	No	
**Variant preference**	***pLess=*[0,1]**	**Yes**	
Increasing conservatism	*d*=0.1,0.2,0.3	No	

Bold fonts indicate conditions in which the sigmoidal model fits the relation between frequency of a behaviour and the probability to copy it better than a linear model, suggesting the population-level pattern of conformist transmission.
